# The new neural pressure support (NPS) mode and the helmet: did we find the dynamic duo?

**DOI:** 10.1186/s44158-024-00170-6

**Published:** 2024-06-10

**Authors:** Alessandro Costa, Federico Merlo, Aline Pagni, Paolo Navalesi, Giacomo Grasselli, Gianmaria Cammarota, Davide Colombo

**Affiliations:** 1 Anesthesia and Intensive Care Unit, ASL Novara, Ospedale SS. Trinità Borgomanero, Novara, Italy; 2 Intensive Care Department, AO Padua, Padua, Italy; 3 Anesthesiology and Intensive Care, Università Degli Studi Di Padova, Padua, Italy; 4 Intensive Care Unit, Fondazione IRCCS Ca’ Granda, Ospedale Maggiore Policlinico Milano, Milan, Italy; 5 Medical, Surgical and Transplant Physiopathology Department, Università Degli Studi Di Milano, Milan, Italy; 6 Anesthesia and Intensive Care Unit, Azienda Ospedaliero, Universitaria SS Antonio E Biagio E Cesare Arrigo Di Alessandria, Alessandria, Italy; 7 Translational Medicine Department, Università Degli Studi del Piemonte Orientale, Novara, Italy; 8 Health Science Department, Università Degli Studi del Piemonte Orientale, Novara, Italy

**Keywords:** Neural pressure support (NPS), Neurally adjusted ventilatory assist (NAVA), Pressure support ventilation (PSV), Patient-ventilator interaction (PVI), Helmet, Noninvasive ventilation (NIV)

## Abstract

**Background:**

Noninvasive ventilation (NIV) is commonly used in clinical practice to reduce intubation times and enhance patient comfort. However, patient-ventilator interaction (PVI) during NIV, particularly with helmet interfaces, can be challenging due to factors such as dead space and compliance. Neurally adjusted ventilatory assist (NAVA) has shown promise in improving PVI during helmet NIV, but limitations remain. A new mode, neural pressure support (NPS), aims to address these limitations by providing synchronized and steep pressurization. This study aims to assess whether NPS per se improves PVI during helmet NIV compared to standard pressure support ventilation (PSV).

**Methods:**

The study included adult patients requiring NIV with a helmet. Patients were randomized into two arms: one starting with NPS and the other with PSV; the initial ventilatory parameters were always set as established by the clinician on duty. Physiological parameters and arterial blood gas analysis were collected during ventilation trials. Expert adjustments to initial ventilator settings were recorded to investigate the impact of the expertise of the clinician as confounding variable. Primary aim was the synchrony time (Time_sync_), i.e., the time during which both the ventilator and the patient (based on the neural signal) are on the inspiratory phase. As secondary aim neural-ventilatory time index (NVT_I_) was also calculated as Time_sync_ divided to the total neural inspiratory time, i.e., the ratio of the neural inspiratory time occupied by Time_sync_.

**Results:**

Twenty-four patients were enrolled, with no study interruptions due to safety concerns. NPS demonstrated significantly longer Time_sync_ (0.64 ± 0.03 s vs. 0.37 ± 0.03 s, *p* < 0.001) and shorter inspiratory delay (0.15 ± 0.01 s vs. 0.35 ± 0.01 s, *p* < 0.001) compared to PSV. NPS also showed better NVT_I_ (78 ± 2% vs. 45 ± 2%, *p* < 0.001). Ventilator parameters were not significantly different between NPS and PSV, except for minor adjustments by the expert clinician.

**Conclusions:**

NPS improves PVI during helmet NIV, as evidenced by longer Time_sync_ and better coupling compared to PSV. Expert adjustments to ventilator settings had minimal impact on PVI. These findings support the use of NPS in enhancing patient-ventilator synchronization and warrant further investigation into its clinical outcomes and applicability across different patient populations and interfaces.

**Trial registration:**

This study was registered on www.clinicaltrials.gov NCT06004206 Registry URL: https://clinicaltrials.gov/study/NCT06004206 on September 08, 2023.

## Introduction

Noninvasive ventilation (NIV) has found various applications in clinical settings, shortening intubation times and preventing orotracheal intubation [1]. NIV success is closely tied to the degree of patient-ventilator interaction (PVI) and the comfort experienced by patients undergoing respiratory assistance [2–4]. During NIV, several factors contribute to the achievement of a good level of PVI and comfort. Among these factors, the interface adopted to deliver NIV plays a crucial role [2]. Notoriously, during NIV, the helmet has been proven to assure more comfort compared to face mask avoiding skin ulcers [2, 5]. In spite of these advantages, NIV delivered through helmet is at high risk of poor PVI due to the high dead space and compliance of the interface, leading to inspiratory and expiratory trigger delay as well as delay in pressurization time [6–9]. All in all, these factors are well-recognized determinants of a scarce patient-ventilator synchrony (PVS), a known cause of premature interruption of NIV [2].

During helmet NIV, neurally adjusted ventilatory assist (NAVA), a ventilatory mode that delivers respiratory support synchronous and proportional to the amount of diaphragmatic activation quantified as electrical activity of the diaphragm (EAdi), has been demonstrated to improve overall PVI, particularly in terms of PVS, and comfort, thanks to the electrically triggered inspiratory assistance [10–12]. Moreover, according to the NAVA setting suggested by Cammarota et al., [9] the adoption of fast pressurization furtherly enhanced PVI and comfort compared to standard NAVA and pressure support ventilation (PSV). On the basis of the previous results [9, 10, 12], a new ventilatory mode, named neural pressure support (NPS), has been recently introduced. NPS exploits all the advantages of the neural (EAdi-piloted) trigger and provides the possibility of applying a steep pressurization during NIV. Thus, NPS should overcome most of the PVI limitations previously described for helmet NIV. The aim of the present study is to determine whether NPS can improve PVS and overall PVI during helmet NIV as compared to standard PSV in critically ill patients requiring NIV assistance regardless the expertise of the clinician who set the ventilator. Our secondary aims are to evaluate the safety of helmet NIV during NPS as compared to PSV, influence of NPS on breathing pattern, and patient-ventilator asynchronies.

## Material and methods

The present investigation was registered on www.clinicaltrials.gov with the number NCT06004206 and approved by the Ethic Committee “Comitato Etico Interaziendale Territoriale” — Novara (IT). The study was conducted in accordance with the “Declaration of Helsinki” principles. All patients gave written informed consent to be included in the study and for data treatment.

### Study protocol

Patients undergoing de novo NIV with the helmet NIMV ZIP (Dimar, Medolla, IT) were included in the study population. Inclusion and exclusion criteria were adult patients (> 18 years of age), noninvasive ventilation indication decided and set by the clinician on duty, nasogastric tube already in place for clinical reasons, and arterial catheter already in place for clinical reasons. Exclusion criteria were as follows: inability to express informed consent, estimated duration of NIV less than 12 h, major gastroesophageal surgery in the last 12 months, gastroesophageal bleeding in the prior month, known history of esophageal varices, recent maxillofacial trauma or surgery, hemodynamic instability despite adequate fluids income (i.e., need of continuous infusion of norepinephrine > 0.1 mcg/kg/min or any continuous infusion of epinephrine to keep systolic arterial pressure > 90 mmHg), temperature > 38 °C, and coagulation disorders (*PT-INR* > 1.5 and/or aPTT ratio > 44 s).

After enrolment, each patient needed to be ventilated for at least 6 h before starting the study protocol to allow proper clinical stability and had a 36-h window to complete the trial.

If not already in place for clinical reasons, a NAVA catheter was inserted, and the patients were connected to a SERVO-U® ventilator (Getinge AB, Solna, SE) equipped with the new NPS® (Getinge AB, Sölna, SE) software. Correct positioning of the NAVA catheter was then verified using the dedicated SERVO-U software as previously described [8], and the EAdi tracing was then hidden from the ventilator screen using the built-in function.

The starting parameters (i.e., inspired oxygen fraction (FiO_2_), positive end expiratory pressure [PEEP], pressure support [PS], inspiratory trigger and expiratory trigger [cycle-off], pressurization time) were unmodified from those set by the clinician on duty according to her/his own judgment. NPS mode was set with the same parameters without any changes from PSV. Additionally, the neural inspiratory trigger was kept at the default value of 0.5 μV, while neural respiratory cycling-off is fixed at 70% of the EAdipeak and cannot be changed by the clinician as described for NAVA ventilation [8].

Enrolled patients were then randomized according to a computer-based algorithm (MedCalc® Statistical Software version 19.4.1 [MedCalc Software Ltd., Ostend, BE]) into two arms: patients in “Track A” started the protocol with 30 min of NPS ventilation, followed by 30 min of PSV, while patients in “Track B” were ventilated in PSV first and then in NPS for the same amount of time. After every 30 min of ventilation, an arterial blood gas (ABG) analysis was performed, and the last 2 min of flow, airways pressure (Paw), and EAdi tracings were recorded using the Servo Tracker SCI® v. 1.1 (Getinge AB, Solna, SE) software at 100 Hz for the analysis.

**Fig. 1:**
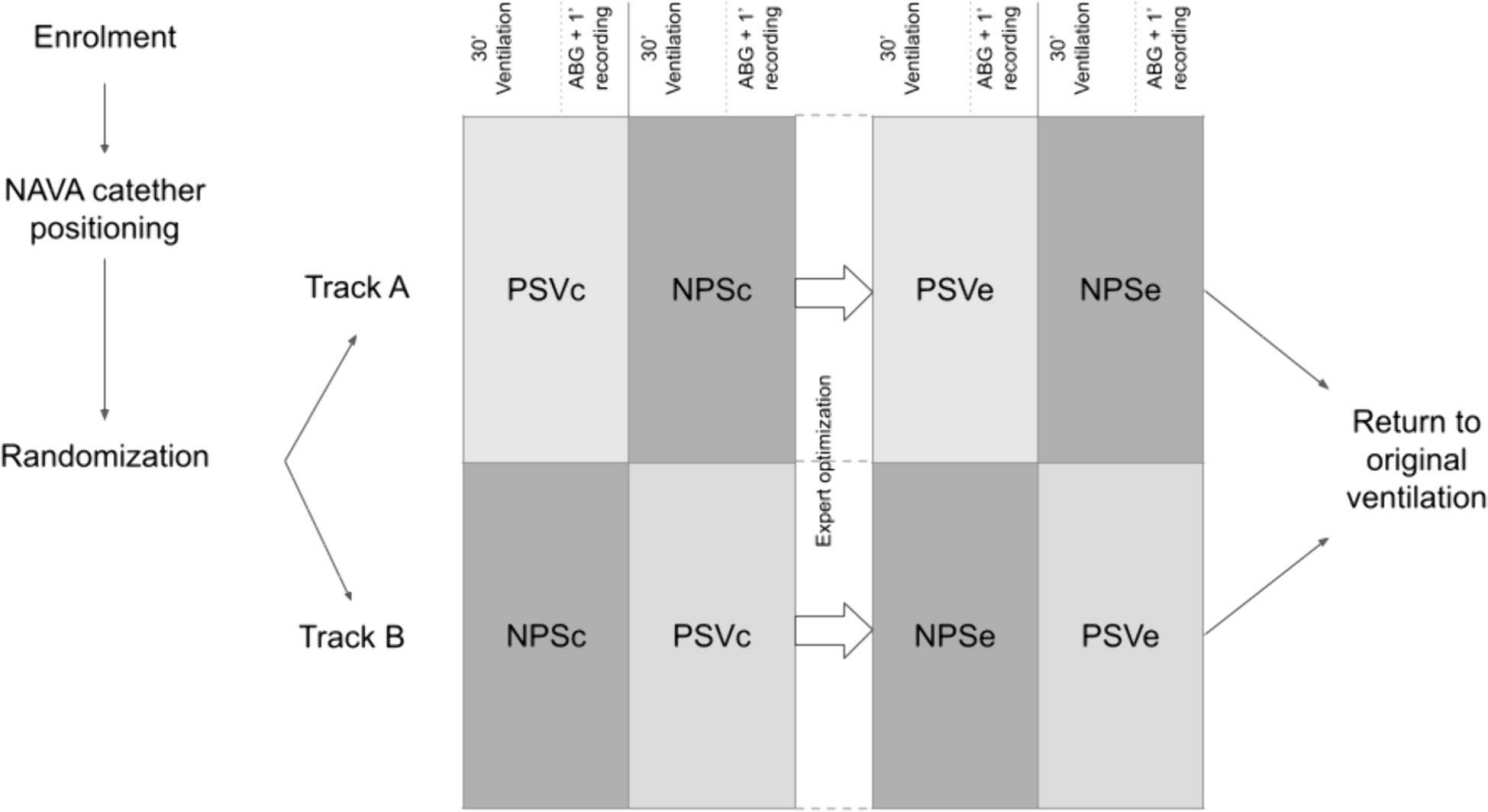
Protocol flowchart including ABGs sampling and waveform recordings at the present time-point

At the end of this first hour of ventilation, a researcher skilled in mechanical ventilation (DC) optimized the ventilatory parameters (PEEP, PS, trigger, cycle-off, and ramp), blinded for the EAdi tracing. After this new setting, the protocol was entirely repeated in the same order assigned by the randomization. Collected data from the ventilator set by the clinician were labeled as NPS_C_ and PSV_C_, while data collected after the expert evaluation were identified as NPS_E_ and PSV_E_. Figure 1 depicts the study flowchart.

The study protocol would have been halted immediately, and the team would have put in place all the appropriate measures to solve the issue at hand if any of the following conditions did occur:

- Tachypnea with *RR* > 40 act/min
- Respiratory distress or dyspnea
- Respiratory acidosis with end-tidal carbon dioxide (EtCO2) increasing over 20% of the baseline level
- Desaturation below 90% with the appropriate FiO2 administered
- Hypotensive or hypertensive events (systolic pressure < 90 mmHg or > 180 mmHg)
- New arrhythmias or tachycardia with HR > 140 bpm
- Neurological alterations or RASS changes (< −3 or > 2)
- Patient discomfort

### Data collection

For every patient enrolled in the study, physiological parameters, reason for admission in ICU, cause for NIV support (i.e., treating acute respiratory failure [ARF] or re-intubation prevention), and the duration of mechanical ventilation before the study enrolment both in invasive and noninvasive ventilation were collected.

Ventilatory parameters collected directly from the ventilator were PEEP, PS, cycle-off, ramp and FiO_2_, and flow-time, pressure–time, and EAdi-time waveform: all these parameters were acquired via Servo Tracker SCI® v1.1 (Getinge AB, Solna, SE).

The following parameters and indexes were extrapolated from the tracks and analyzed in a semiautomated mode via ICU Lab software (KleisTEK, Bari, IT):

- Mechanical respiratory rate (RRmech) (breath/min): Respiratory rate measured on the flow track
- Neural respiratory rate (RRneu) (breath/min): Respiratory rate measured on the EAdi
- Expiratory tidal volume (VTexp) (l): Expiratory tidal volume
- Leaked tidal expiratory volume (VTleak) (l): Leaked volume for each respiratory act
- Synchrony time (TimeSync) (s): Defined as the time during which both EAdi and flow tracings are in the inspiratory phase, meaning the patient is willing to inspirate and the ventilator is providing support.
- Inspiratory delay (DelayInsp) (s): Defined as the time between the beginning of the neural inspiration and the time where the pressure curve turned positive
- Asynchrony index (AI): Ratio between the number of ineffective efforts, auto-triggers and double triggers, and the neural respiratory rate plus auto-triggers
- Neural-ventilatory time index (NVTI): Calculated as TimeSync/TIneu
- Peak of EAdi (EAdiPeak) (μV): The maximum EAdi measured during inspiration

ABG data, collected after each round of ventilation, comprised pH, PaO2, paCO2, HCO3, lactate, and P/F.

### Statistical analysis

The primary endpoint of the study was Time_synch_.

On the basis of a previous study [13], considering a difference between means for a two-way ANOVA of at least 0.25 s (two groups, four measurements), with an alpha error of 0.05 and a beta error of 0.20, the sample size resulted to 24 patients (G*Power 3.1.9.6, Heinrich-Heine-Universität, Düsseldorf, DE).

In order to avoid carry-on effect, the starting ventilation was randomized (Track A or track B) using a random sequence with two variables, generated via MedCalc Statistical Software version 19.4.1 (MedCalc Software Ltd., Ostend, Belgium).

Population distribution will be verified using the Kolmogorov–Smirnov test.

Continuous data will be analyzed considering the effect and the interaction of both the ventilation mode and the settings adjustment by the expert of mechanical ventilation via the two-way ANOVA for repeated measures test.

The null hypothesis will be rejected for *p* < 0.05.

The software used for the statistical analysis is MedCalc Statistical Software version 19.4.1 (MedCalc Software Ltd., Ostend, Belgium).

## Results

All results will be presented in mean ± standard deviation.

### Population

**Fig. 2:**
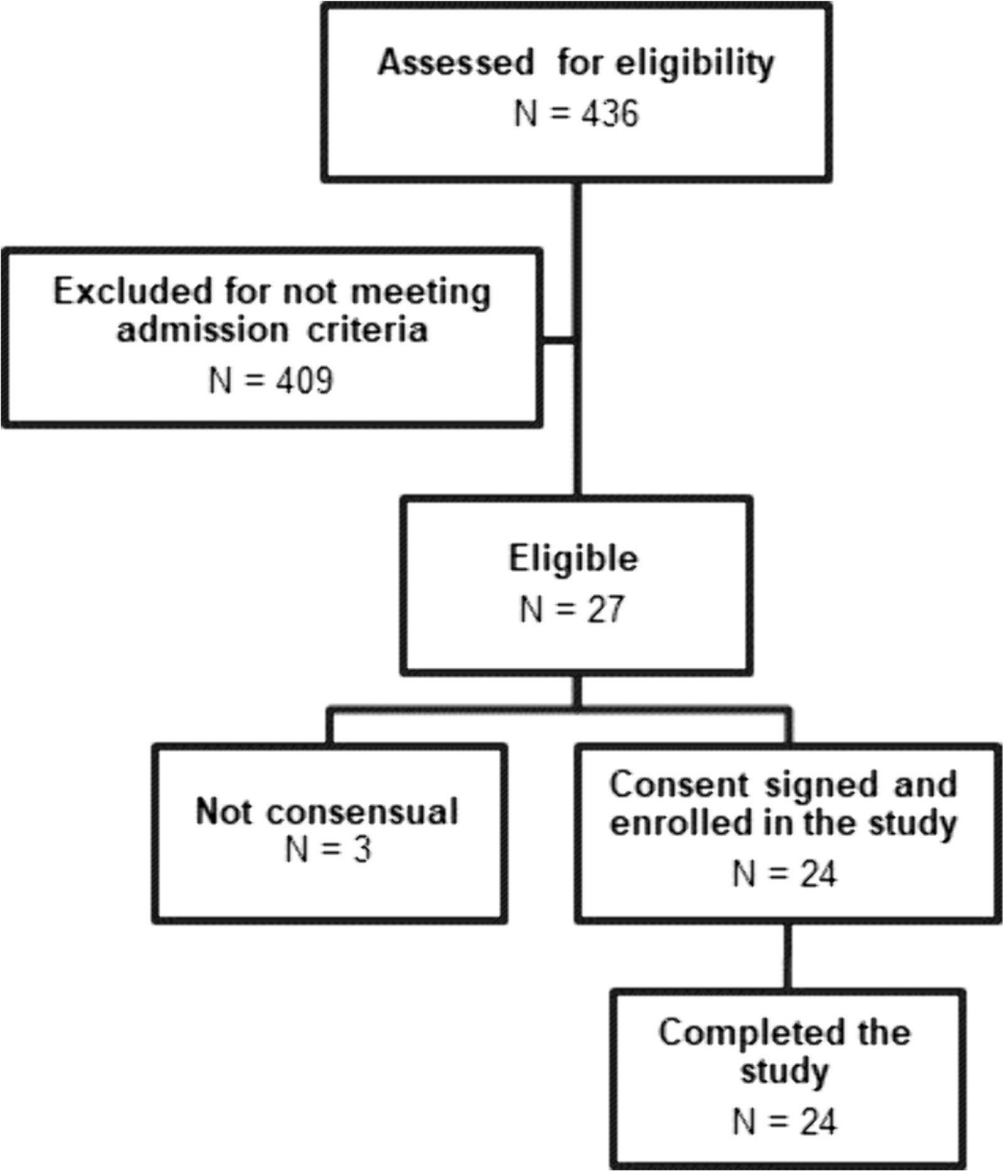
CONSORT diagram of patient screening and enrolment

All the patients admitted to ICU (April 2022-June 2023) during the enrolment phase of the study unit were screened for eligibility. Of the 27 eligible patients, 24 consented to be enrolled in the study. No patient interrupted the study due to the safety criteria (Fig. 2).

Mean age was 68.3 ± 13.5 years, and BMI was 28.4 ± 6.8. Forty-two percent of the enrolled was male and 58% female.

Admission diagnosis was ARDS for six of them; five had a septic shock; four had COPD; two required treatment for allergic asthma, pneumonia, sepsis, or acute respiratory insufficiency (ARF) secondary to a motoneuronal disease (MND); and one had a status epilepticus. Eight patients required NIV for the treatment of hypoxia, while 16 required NIV for prevention of reintubation.

Before enrolment, mean mechanical ventilation was 6.9 ± 7.1 days long, while time spent in NIV before enrolment was 31.2 ± 18.6 h (Table 1).

**Table 1:** Demographic data of enrolled population with randomization, age, gender, body mass index (BMI), diagnosis for admission (admission), indication for NIV (acute respiratory failure; prevention of re-intubation), and duration of mechanical ventilation both invasive and noninvasive before enrollment

**N**	**Group**	**Age** (y)	**Gender** (M; F)	**BMI** (kg/m^2^)	**Admission**	**ARF**	**Prevention of reintubation**	**Invasive ventilation before enrolment** (days)	**NIV before enrolment** (h)
1	B	50	M	31.2	ARDS		x	15	29
2	B	63	M	35.3	Sepsis	x		0.5	12
3	A	80	F	24.6	Pneumonia		x	4	24
4	A	62	M	23.9	Status epilepticus		x	6	24
5	A	72	M	22.5	COPD		x	2	28
6	B	62	F	17.5	COPD	x		2	24
7	B	74	F	27.5	ARDS		x	1	21
8	B	25	M	29.8	ARDS		x	6	10
9	A	73	F	23.8	ARF from MND		x	5	20
10	A	72	M	16.3	ARDS		x	7	72
11	B	71	F	39.6	ARF from MND		x	3	6
12	B	81	M	29.4	Septic shock		x	29	36
13	B	56	M	22.3	Pneumonia	x		1	24
14	B	72	F	41.1	Septic shock		x	13	24
15	A	71	F	23.4	COPD	x		3	72
16	B	43	M	25.9	Allergic asthma		x	20	48
17	A	80	F	26.6	Sepsis	x		2.5	50
18	B	70	M	29.3	ARDS		x	15	60
19	A	71	F	39.7	Septic shock		x	9	48
20	A	81	F	37.5	Septic shock		x	3	6
21	A	77	F	36.3	Septic shock		x	3	48
22	A	72	F	24.1	Allergic asthma	x		5	22
23	A	81	F	20.7	ARDS	x		9	16
24	B	80	F	22.2	COPD	x		1	24

#### Patient-ventilator interaction

**Fig. 3:**
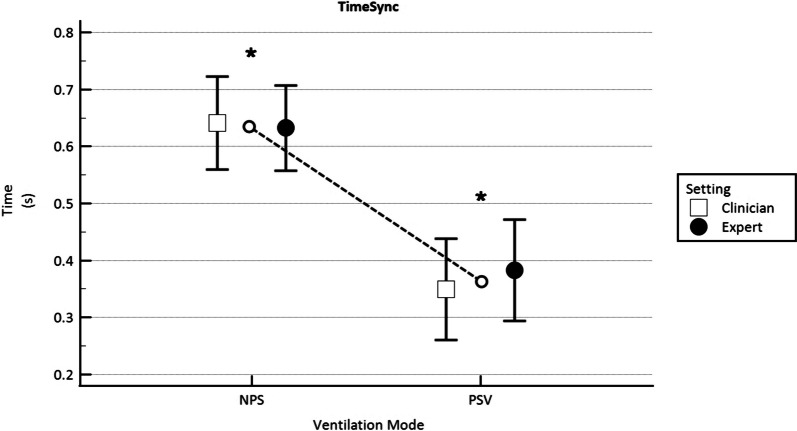
Synchrony time (Time_Sync_) compared in NPS and in PSV mode set by the clinician (C = hollow square) and the expert (E = filled circle), expressed in seconds (s). The hollow circles joined by the dotted line represent the average value between C and E mean values. *p < 0.001 for differences between NPS and PSV

Time_Sync_ was longer in NPS 0.64 ± 0.03 s as compared to PSV 0.37 ± 0.03 (*p* < 0.001), regardless of the expertise of the clinician who set the ventilator (*p* = 0.76), as can be seen in Fig. 3.

**Fig. 4:**
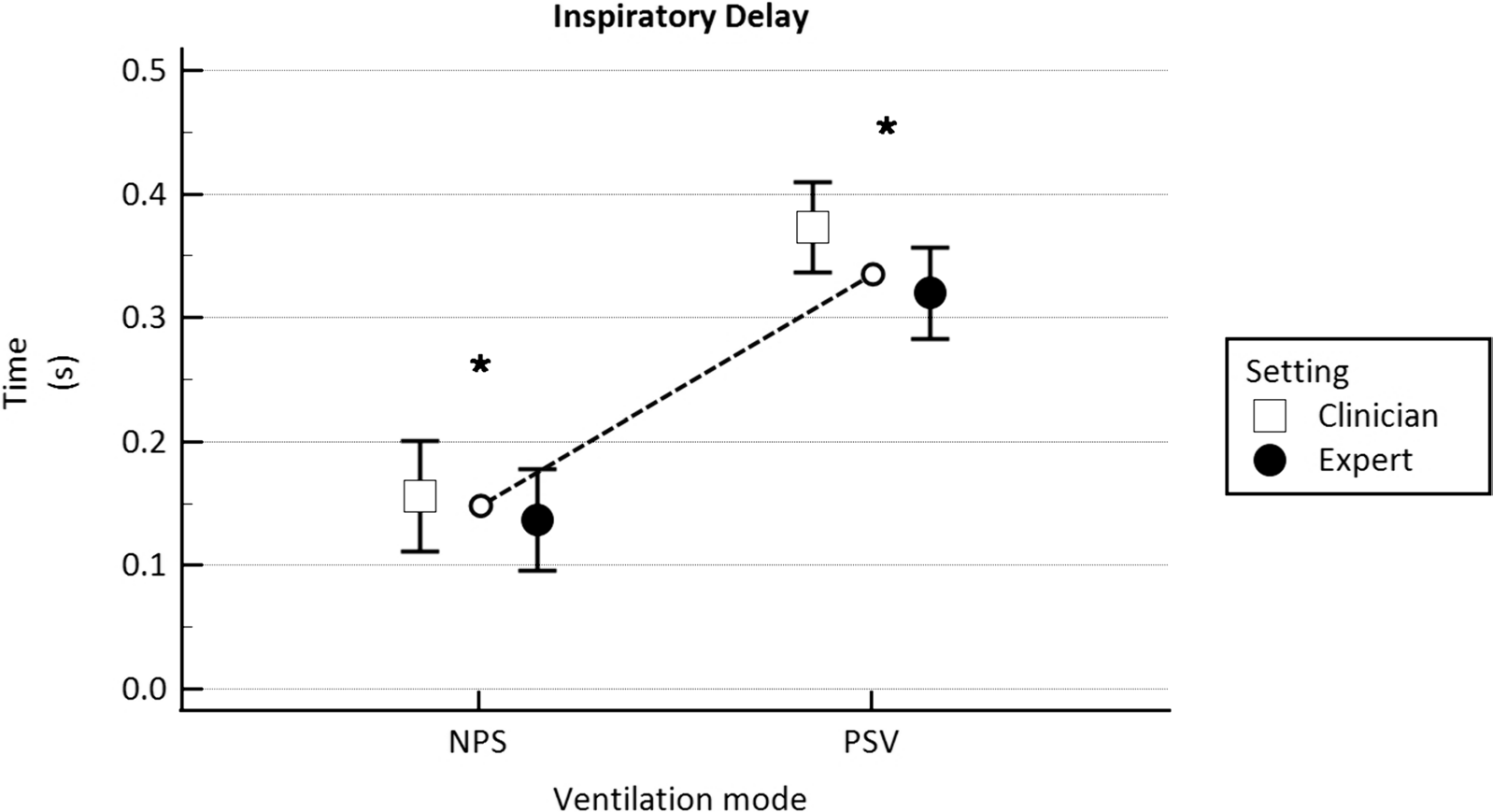
Inspiratory delay compared in NPS and in PSV mode set by the clinician (C = hollow square) and the expert (E = filled circle), expressed in seconds (s). The hollow circles joined by the dotted line represent the average value between C and E mean values. *p < 0.001 for differences between NPS and PSV

Inspiratory delay was found statistically different between *NPS* 0.15 ± 0.01 s and *PSV* 0.35 ± 0.01 s (*p* < 0.001) with a shorter time in NPS, with no significant differences according to the expertise of the clinician who set the ventilator (*p* = 0.06), as reported in Fig. 4.

**Fig. 5:**
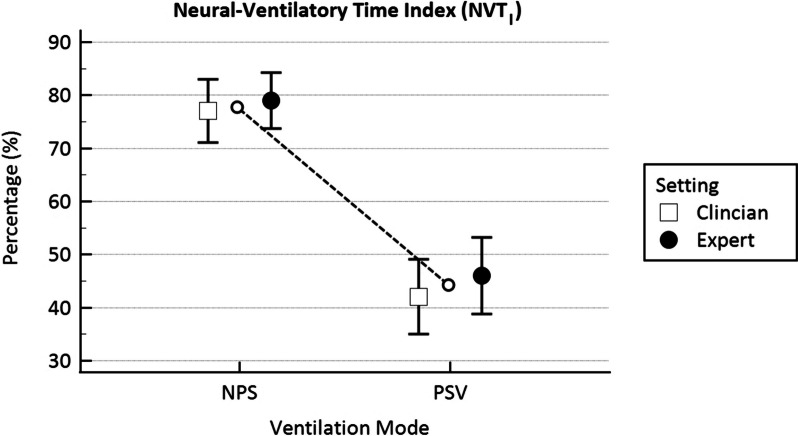
Neural-ventilatory time index (NVT_I_) compared in NPS and in PSV mode set by the clinician (C = hollow square) and the expert (E = filled circle), calculated as Time_Sync_/TI_neu_ and expressed in percentage (%). The hollow circles joined by the dotted line represent the average value between C and E mean values. *p < 0.001 for differences between NPS and PSV

The NVT_I_ was statistically different between NPS 78 ± 2% and PSV 45 ± 2% (*p* < 0.001), showing a better coupling in NPS regardless of the expertise of the clinician who set the ventilator (*p* = 0.38), as shown in Fig. 5.

AI was not statistically different between both NPS vs. PSV (*p* = 0.93) and C vs. E (*p* = 0.80). In each group, its value was < 10%.

#### Measured ventilation data

The analysis of the EAdi_Peak_, RR_mec_, RR_neu_, VT_exp_, and VT_leak_ is shown in Table 2.

**Table 2:** EAdipeak, respiratory rates, and tidal volume. Data presented as mean ± standard deviation

	**PSV**_**c**_	**NPS**_**c**_	**PSV**_**e**_	**NPS**_**e**_	***p***
**EAdi**_**Peak**_ (μV)	35.73 ± 23.28	31.8 ± 23.03	30.13 ± 19.51	30.4 ± 18.26	PSV vs NPS = 0.629 C vs E = 0.43
**RR**_**mech**_ (act/min)	24.17 ± 6.29	24.15 ± 6.24	26.98 ± 6.12	24.94 ± 4.89	PSV vs NPS = 0.395 C vs E = 0.139
**RR**_**neu**_ (act//min)	22.92 ± 6.61	23.50 ± 6.99	25.02 ± 6.27	24.33 ± 5.52	PSV vs NPS = 0.894 C vs E = 0.706
**VT**_**exp**_ (L)	0.88 ± 0.76	1.09 ± 0.93	0.78 ± 0.4	0.89 ± 0.36	PSV vs NPS = 0.231 C vs E = 0.259
**VT**_**leak**_ (L)^a^	0.13 ± 0.64	0.36 ± 0.86	− 0.11 ± 0.32	0.15 ± 0.3	PSV vs NPS = 0.242 C vs E = 0.338

^a^Leak is calculated as VTinsp-VTexp

No statistical differences were found in any of these variables, nor according to the ventilation mode and nor according to the expertise of the clinician who set the ventilator.

### ABGs

ABGs variables did not change with statistical significance in any of the four trials tested. Table 3 depicts the results of each trial and corresponding *p*-level both for NPS vs. PSV and C vs. E.

**Table 3:** Arterial blood gas data. Data presented as mean ± standard deviation

	**PSV**_**c**_	**NPS**_**c**_	**PSV**_**e**_	**NPS**_**e**_	***p***
**pH**	7.45 ± 0.05	7.44 ± 0.05	7.45 ± 0.05	7.45 ± 0.06	PSV vs NPS = 0. 89 C vs E = 0.998
**PaO**_**2**_ (mmHg)	86.07 ± 15.07	84.66 ± 14.37	93.06 ± 18.13	89.97 ± 17.69	PSV vs NPS = 0.508 C vs E = 0.072
**PaCO**_**2**_ (mmHg)	47.42 ± 12.9	47.71 ± 13.16	47.14 ± 13.12	47.49 ± 13.68	PSV vs NPS = 0.906 C vs E = 0.927
**p/F**	249.17 ± 66.01	247.88 ± 72.07	266.91 ± 62.85	257.22 ± 63.79	PSV vs NPS = 0.689 C vs E = 0.325
**HCO**_**3**_ (mmol/L)	32.38 ± 1.48	32.8 ± 1.48	32.36 ± 1.51	32.66 ± 1.51	PSV vs NPS = 0.809 C vs E = 0.957
**Lac** (mmol/L)	1.12 ± 0.12	1.12 ± 0.12	1.12 ± 0.13	1.16 ± 0.13	PSV vs NPS = 0.863 C vs E = 0.884

### Ventilator parameters

According to the protocol, no differences were found between PSV and NPS analyzing any of the ventilation parameters (i.e., PEEP, PS, cycle-off, ramp, and FiO_2_ [*p* = 1]). The expert clinician set did not affect PS and FiO_2_, while it resulted statistically different for PEEP, ramp, and cycle-off. Specifically, the expert intervention resulted in an overall slight, although significant, increase in PEEP level (10.0 ± 0.15 cmH2O vs. 10.5 ± 0.15 cmH_2_O for C vs. E, respectively [*p* = 0.02]), a faster cycle-off (44.4 ± 1.9% vs. 50.4 ± 1.9% for C vs. E, respectively [*p* = 0.02]), and a more rapid pressurization time (i.e., ramp) (0.11 ± 0.01 s vs. 0.02 ± 0.01 s for C vs. E, respectively [*p* < 0.0001]).

## Discussion

Time_synch_ resulted longer in NPS than in PSV, while the parameters set by the expert did not affect Time_synch_. This may confirm the hypothesis that NPS is, per se, able to provide a better PVI when used with helmet NIV. These data are consistent with findings from a previous empirical study from Cammarota et al. [9] and open new frontiers in the use of helmet NIV. During NPS, the patient’s inspiratory effort was effectively supported for more than 75% of the time, while during PSV, it occurred for an amount of time lower than 50%, according to NVT_I_. In another previous empirical study, utilizing the so-called NAVA15, Longhini et al. [8] found similar results, concluding that with NAVA15 settings, helmet NIV had performance in terms of P–V synchrony equal to PSV face mask NIV but higher comfort [8]. Helmet is suggested as the most comfortable interface for NIV delivery in several studies [8, 14, 15]. This might be explained by the less occurrence in air leak [14] and the possibility to feed the patient [15]. Unfortunately, the helmet dead space and compliance impact negatively on synchronism and pressurization [13], resulting in an overall less efficient device. Our findings suggest that NPS may be effectively used to overcome these limitations.

According to these findings, the main responsible for the synchronism improvement is the reduction in the Delay_Insp_, which in NPS is around 200 ms faster than in PSV. Notably, the significant reduction of the pressurization time set by the expert physician had only a minor effect (C vs. E *p* = 0.06) on the overall reduction of Delay_Insp_. Conversely, the cycling-off is mainly affected from the expertise of the physician who set the ventilator. This might be explained by the fact that the cycling-off is not fully dependent on an algorithm following the principle “first come, first served” as it occurs for the Trigger_Insp_ [7]. Since during PSV the setting of the cycling-off criteria plays a major role, it is not surprising that an expert physician is still able to improve that phase even during a neural-driven ventilation. Despite being statistically significant, the change in PEEP level had quite low clinical relevance since it is of an amount less than 1 cmH_2_O. No other differences were found in the ventilator setting for PS and FiO_2_. Overall, the other breathing pattern parameters seem to be not affected by both mode of ventilation and ventilator settings: nevertheless, although not significant, the only mode during which RR_mech_ and RR_neu_ resulted equal was NPS_e_ (Table 2). No differences were found with respect to EAdi_peak_ all along the four trials. In the study of Cammarota et al. [9], EAdi_peak_ resulted lower by using ventilator settings mimicking the NPS mode. The different sedative drugs and level [16, 17] may play a role in patient’s effort. Not least in the study of Cammarota [9], the PS was kept constant for all the patients and modes around 10 cmH_2_O (per protocol); in our study, the PS level was set by the clinician on duty according to patient’s estimated requirement, and so far, it is quite difficult to compare the results of these studies. ABGs data resulted stable during all the study in all modes and settings (Table 4).

**Table 4:** Ventilation parameters

	**PSV**_**c**_	**NPS**_**c**_	**PSV**_**e**_	**NPS**_**e**_	***p***
**PEEP** (cmH_2_O)	10.04 ± 1.27	10.04 ± 1.27	10.54 ± 0.83	10.54 ± 0.83	PSV vs. NPS = 1 **C vs. E = 0.025**
**PS** (cmH_2_O)	6.83 ± 2.5	6.83 ± 2.5	6.33 ± 2.44	6.33 ± 2.44	PSV vs. NPS = 1 C vs. E = 0.324
**Cycle-off** (%)	44.38 ± 2.64	44.38 ± 2.64	50.42 ± 2.64	50.42 ± 2.64	PSV vs. NPS = 1 **C vs. E = 0.025**
**Ramp** (s)	0.11 ± 0.10	0.11 ± 0.10	0.02 ± 0.05	0.02 ± 0.06	PSV vs. NPS = 1 **C vs. E < 0.001**
**FiO**_**2**_ (%)	36.04 ± 8.07	36.04 ± 8.07	36.04 ± 8.07	36.04 ± 8.07	PSV vs. NPS = 1 C vs. E = 1

This study has some limitations: it is as a single-center study on a limited number of patients, it may be not representative of the general ICU practice. Moreover, in our ICU, the staff is highly skilled in handling noninvasive respiratory support overall and helmet NIV in particular; thus, differences between NPS and PSV during helmet NIV may differ according to expertise of the staff both with interface and NIV practice.

## Conclusion

This physiological study confirms that NPS is safe and improves P–V interaction and synchronization when used with helmet NIV. Further studies will be necessary to evaluate the effect of this new mode on patient’s outcomes and its effectiveness on different interfaces as well as on intubated patients.

## Data Availability

The present investigation was registered on www.clinicaltrials.gov with the number NCT06004206 on September 08, 2023. The Ethic Committee “Comitato Etico Interaziendale Territoriale” - Novara (IT) approved the study.
